# Cross-disease analysis of Alzheimer’s disease and type-2 Diabetes highlights the role of autophagy in the pathophysiology of two highly comorbid diseases

**DOI:** 10.1038/s41598-019-39828-5

**Published:** 2019-03-08

**Authors:** Laura Caberlotto, T.-Phuong Nguyen, Mario Lauria, Corrado Priami, Roberto Rimondini, Silvia Maioli, Angel Cedazo-Minguez, Giulia Sita, Fabiana Morroni, Mauro Corsi, Lucia Carboni

**Affiliations:** 1 The Microsoft Research, University of Trento Centre for Computational Systems Biology (COSBI), Rovereto, Italy; 2 Life Sciences Research Unit, University of Luxembourg, Esch-sur-Alzette, Luxembourg; 3 Department of Mathematics, University of Trento, Povo, Trento Italy; 4 Department of Medical and Surgical Science, Alma Mater Studiorum University of Bologna, Bologna, Italy; 5 Department of Neurobiology, Care Sciences and Society, Division of Neurogeriatrics, Karolinska Institutet, Stockholm, Sweden; 6 Department of Pharmacy and Biotechnology, Alma Mater Studiorum University of Bologna, Bologna, Italy; 7 Aptuit, an Evotec company, Drug Design and Discovery, Verona, Italy; 8 Present Address: Aptuit an Evotec company Drug Design and Discovery, Verona, Italy; 9 Present Address: Megeno S.A.6A, avenue des Hauts-FourneauxL-4362 Esch-sur-Alzette, Esch-sur-Alzette, Luxembourg

## Abstract

Evidence is accumulating that the main chronic diseases of aging Alzheimer’s disease (AD) and type-2 diabetes mellitus (T2DM) share common pathophysiological mechanisms. This study aimed at applying systems biology approaches to increase the knowledge of the shared molecular pathways underpinnings of AD and T2DM. We analysed transcriptomic data of post-mortem AD and T2DM human brains to obtain disease signatures of AD and T2DM and combined them with protein-protein interaction information to construct two disease-specific networks. The overlapping AD/T2DM network proteins were then used to extract the most representative Gene Ontology biological process terms. The expression of genes identified as relevant was studied in two AD models, 3xTg-AD and ApoE3/ApoE4 targeted replacement mice. The present transcriptomic data analysis revealed a principal role for autophagy in the molecular basis of both AD and T2DM. Our experimental validation in mouse AD models confirmed the role of autophagy-related genes. Among modulated genes, Cyclin-Dependent Kinase Inhibitor 1B, Autophagy Related 16-Like 2, and insulin were highlighted. In conclusion, the present investigation revealed autophagy as the central dys-regulated pathway in highly co-morbid diseases such as AD and T2DM allowing the identification of specific genes potentially involved in disease pathophysiology which could become novel targets for therapeutic intervention.

## Introduction

Alzheimer’s disease (AD) is the most common cause of dementia and it is characterized by histopathological, molecular, and biochemical abnormalities, including cell loss; abundant neurofibrillary tangles; dystrophic neurites; amyloid-β (APP-Aβ) deposits; impaired energy metabolism; mitochondrial dysfunction; chronic oxidative stress; and DNA damage^1^.

While the cause of AD remains unknown, several risk factors have been identified that may provide insight into the fundamentals of AD pathogenesis. Among them, aging is by far the most important risk factor, but type-2 diabetes mellitus (T2DM) is also a known risk factor for AD. Compelling evidence supports the notion that insulin deficiency and insulin resistance are involved in AD-type neurodegeneration; (1) increased risk of developing mild cognitive impairment (MCI), dementia, or AD in individuals with T2DM^2,3^ (2) progressive brain insulin resistance, insulin deficiency in AD^4–7^; (3) cognitive impairment in experimental animal models of T2DM^8^ (4) improved cognitive performance in experimental models and humans with AD or MCI after treatment with insulin sensitizer agents or intranasal insulin^9–16^ (5) shared molecular, biochemical, and mechanistic abnormalities in T2DM and AD^2,17,18^. Based on the concept that AD may represent a brain-specific form of diabetes mellitus, the term “type-3 diabetes” indicating AD was coined^4,19^.

Cross-disease therapeutic potential of these major chronic diseases of aging is already showing promise as intranasal insulin improves cognitive performances in patients with mild cognitive impairment and AD^20^. Moreover, new generation anti-diabetic incretin drugs show efficacy in AD animal models^21,22^, and are presently being evaluated in clinical trials in patients, as well as thiazolidinedione antidiabetic agents^23^.

**Figure 1:**
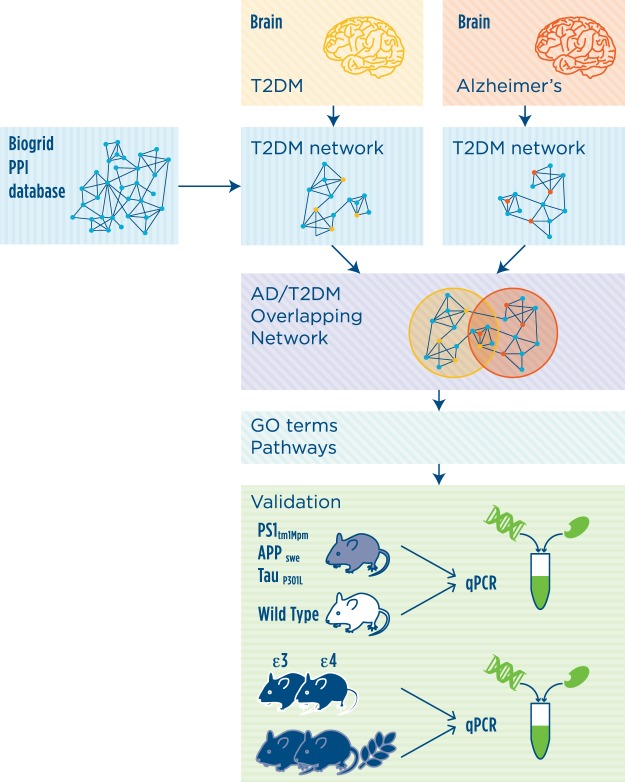
Schematic representation of experimental design. The transcriptional signature associated to AD or T2DM was extracted from transcriptomic data of post-mortem cerebral cortices of AD or T2DM affected individuals as compared to controls. Network analysis was then performed with the reference protein-protein interaction (PPI) network derived from Biogrid data. The functionality analysis of the networks was then performed by testing over-represented Gene Ontology biological process terms and pathways. The relevance of autophagy, the most significant pathway identified by network analysis, was further validated in two animal models of AD.

Systems biology has been paving the way to the exploration of complex associations of diseases and, thus, to the inference of the pathogenic mechanism of a particular disease by considering disease-related components in a large-scale network. Thus, in the present study we set to determine the similarities and differences in the molecular mechanistic networks underlying brain T2DM pathophysiology in AD and in neurologically normal subjects. A novel approach to the analysis of existing transcriptional data-sets of T2DM and AD patient cerebral cortex material was used, with a major application to human brain networks (Fig. 1). This integration of knowledge highlighted a central role for the autophagy pathway in the mechanisms underlying the commonalities in AD and T2DM, which was further analyzed in animal models of disease. We focus our investigation initially on human diseased subjects considering the limitation of animal models of CNS-related diseases in representing all aspects of a complex multifactorial disease, but we were also able to confirm and investigate further the molecular mechanism found to be altered in two models that represent different aspects of the genetic of AD.

These findings contribute to opening new ways to tackle some of the more important challenges to combat these disorders.

## Results

### Network analysis of human transcriptomic data

We extracted the following transcriptomic profiles from the GSE36980 dataset: T2DM controls (n = 20), non-T2DM controls (n = 12), T2DM AD (n = 6), and non-T2DM AD subjects (n = 19). Having obtained four well defined groups representing all combinations of our two phenotypes of interest (T2DM and AD), we sought to characterize them transcriptionally in a highly specific way, in the sense of identifying genes whose expression changes were most related to a single phenotype. We derived the set of fold changes profiles by dividing each original profile by a reference one obtained as the genewise average across the whole dataset.

We then applied a rank-based signature extraction algorithm that characterizes each sample by the list of genes with the most extremes fold changes in the corresponding profile. As part of the algorithm, we measured the degree of similarity between each pair of signatures, and we used the resulting similarity matrix to plot a map of the samples and their relative similarity (Supplementary Figure 1). We found that it was necessary to perform a preliminary feature selection step in order to preferentially steer the algorithm toward brain transcriptional alterations that have some association to T2DM and AD, reducing the influence of other factors such age, sex, tissue of origin. We empirically determined that the best results in terms of separation of the groups were obtained by filtering genes whose fold changes showed a sufficiently large difference between average T2DM and non-T2DM values in either the AD or the non-AD cohort (t-test, p-value < 0.01). Despite having based the selection on T2DM status, we noticed that the selected features (N = 483) included a sufficient number of genes differentially expressed between AD and non-AD profiles (N = 103 out of 483).

Having identified list of signature genes capable of separating each of the four group from the others to a satisfactory degree (Supplementary Figure 1), we proceeded to select two of the groups (T2DM controls and non-T2DM AD subjects) for a more detailed analysis. The consensus transcriptional signature for each of these two groups was extracted using an algorithm that summarizes the level of popularity of signature genes across the signatures of that group.

The resulting T2DM transcriptional signature consisted of 126 genes, while the AD signature was composed of 108 genes (Supplementary Table S1).

The transcriptional signatures were computed to be the shortest possible while maintaining high classification accuracy. Therefore, to expand the comprehension of the pathophysiological processes underlying the investigated diseases, we enriched the lists of signature genes by means of a network analysis technique leveraging available knowledge on human protein-protein interactions. Following enrichment, the AD network included 620 genes and 889 interactions, while the T2DM network included 641 genes and 869 interactions. Hence, we obtained an overlapping AD/T2DM network formed by 158 genes in common between the AD and T2DM networks (Supplementary Table S1). The overlapping AD/T2DM network is highly enriched in genes supporting the common neurobiological dysregulations of the two diseases.

### Functional annotation analysis

The functionality analysis of the networks was then performed by testing over-represented Gene Ontology biological process terms and pathways of the signature, the overlapping genes between signatures, AD and T2DM networks separately and overlapping genes between these two networks.

The analysis of the AD network GO biological processes terms revealed a predominant role of Neurotrophin and Notch signalling, apoptosis, autophagy and inflammatory pathways (Supplementary Table 1). Similar findings were confirmed by the pathway analysis, with the addition of Wnt, estrogen, prolactin and FoxO signalling pathways (Table 1).

**Table 1:** Results of the pathway analysis of the AD network. In bold the pathways in common between the AD and T2DM networks.

p value	q value	Pathway	Source
6.75E-13	1.40E-11	Ubiquitin mediated proteolysis - Homo sapiens (human)	KEGG
**5.04E-10**	**8.57E-09**	**NOTCH1 Intracellular Domain Regulates Transcription**	**Reactome**
2.06E-09	3.24E-08	Adaptive Immune System	Reactome
**7.35E-08**	**7.91E-07**	**Signaling by NOTCH**	**Reactome**
2.91E-07	2.95E-06	HDACs deacetylate histones	Reactome
**3.16E-06**	**2.58E-05**	**FoxO signaling pathway - Homo sapiens (human)**	**KEGG**
**3.20E-05**	**0.00022031**	**Macroautophagy**	**Reactome**
**4.16E-05**	**0.00027593**	**Regulation of autophagy - Homo sapiens (human)**	**KEGG**
4.57E-05	0.00028679	Oxidative Stress Induced Senescence	Reactome
0.0005419	0.00254964	Activation of NF-kappaB in B cells	Reactome
**0.0006026**	**0.0027859**	**Wnt signaling pathway - Homo sapiens (human)**	**KEGG**
0.0007463	0.00336957	Notch signaling pathway - Homo sapiens (human)	KEGG
**0.0009247**	**0.004095**	**Neurotrophin signaling pathway - Homo sapiens (human)**	**KEGG**
0.0018044	0.00728684	Amyloids	Reactome
0.0018537	0.00745715	Cytokine Signaling in Immune system	Reactome
0.0022241	0.00861836	Rap1 signalling	Reactome
0.002268	0.00872443	Estrogen signaling pathway - Homo sapiens (human)	KEGG
0.0029791	0.01113449	Chemokine signaling pathway - Homo sapiens (human)	KEGG
0.0032317	0.01194569	Prolactin signaling pathway - Homo sapiens (human)	KEGG
**0.0050714**	**0.01686197**	**Regulation of lipid metabolism by Peroxisome proliferator-activated receptor alpha (PPARalpha)**	**Reactome**
**0.0055406**	**0.01830646**	**Signaling by Interleukins**	**Reactome**
0.0079904	0.02504608	Intrinsic Pathway for Apoptosis	Reactome
0.0088872	0.02728608	Interferon Signaling	Reactome

The T2DM network was characterized by apoptosis and several metabolic-associated processes including lipid, carbohydrate and ketone metabolism, cholesterol homeostasis, and autophagy. Moreover, in the T2DM network, signalling pathways such as neurotrophin, Notch and Wnt were enriched and, as for the AD network, inflammatory pathways were highlighted (Supplementary Table 1). Pathway analysis was in line with the GO terms investigation, with the addition of pathways related to insulin, leptin, prolactin, mTOR, ephrin signalling, and circadian rhythmicity (Table 2).

**Table 2:** Results of the pathway analysis of the T2DM network. In blue the pathways in common between the AD and T2DM networks.

p value	q value	Pathway	Source
3.74E-15	6.64E-13	Thyroid hormone signaling pathway - Homo sapiens (human)	KEGG
1.17E-13	1.38E-11	mechanism of gene regulation by peroxisome proliferators via ppara	BioCarta
**2.79E-11**	**2.29E-09**	**FoxO signaling pathway - Homo sapiens (human)**	**KEGG**
**1.28E-10**	**8.49E-09**	**Macroautophagy**	**Reactome**
**1.44E-10**	**9.05E-09**	**NOTCH1 Intracellular Domain Regulates Transcription**	**Reactome**
**3.31E-09**	**1.14E-07**	**Beta-catenin phosphorylation cascade**	**Reactome**
**4.50E-09**	**1.41E-07**	**Regulation of lipid metabolism by Peroxisome proliferator-activated receptor alpha (PPARalpha)**	**Reactome**
**6.66E-09**	**1.97E-07**	**Circadian Clock**	**Reactome**
**1.15E-08**	**2.98E-07**	**Signaling by Interleukins**	**Reactome**
1.71E-08	4.05E-07	Circadian rhythm - Homo sapiens (human)	KEGG
2.04E-08	4.17E-07	Signaling by Type 1 Insulin-like Growth Factor 1 Receptor (IGF1R)	Reactome
2.47E-08	4.77E-07	AMPK signaling pathway - Homo sapiens (human)	KEGG
3.91E-08	7.06E-07	IRS-mediated signalling	Reactome
5.72E-08	9.46E-07	Insulin receptor signalling cascade	Reactome
2.19E-07	2.72E-06	PI3K-Akt signaling pathway - Homo sapiens (human)	KEGG
3.70E-07	4.19E-06	Signaling by Wnt	Reactome
**9.83E-07**	**9.35E-06**	**Signaling by NOTCH**	**Reactome**
2.77E-06	2.46E-05	Signaling by Leptin	Reactome
4.19E-06	3.46E-05	Adipocytokine signaling pathway - Homo sapiens (human)	KEGG
4.89E-06	3.88E-05	Fatty acid, triacylglycerol, and ketone body metabolism	Reactome
2.94E-05	0.000165458	Sphingolipid signaling pathway - Homo sapiens (human)	KEGG
**0.000475166**	**0.001648377**	**Wnt signaling pathway - Homo sapiens (human)**	**KEGG**
0.000531621	0.001820503	mTOR signalling	Reactome
0.001563324	0.004637717	Cellular Senescence	Reactome
**0.001694211**	**0.004998158**	**Neurotrophin signaling pathway - Homo sapiens (human)**	**KEGG**
0.003142557	0.008559651	EPH-Ephrin signaling	Reactome
0.004303806	0.011317415	Interleukin-6 signaling	Reactome
0.006542263	0.015980527	Insulin signaling pathway - Homo sapiens (human)	KEGG
0.008386082	0.019505104	Prolactin receptor signaling	Reactome

**Figure 2:**
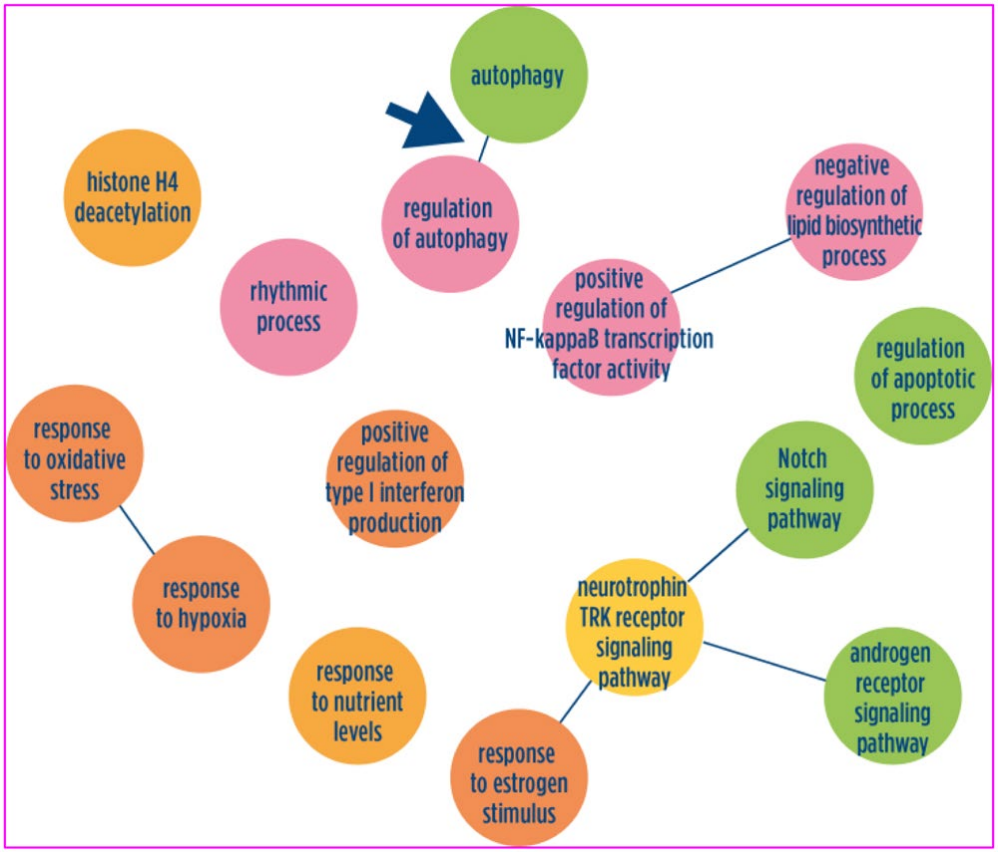
Schematic graphs of over-represented Gene Ontology biological process terms in AD/T2DM overlapping network. Over-represented GO terms in the AD/T2DM overlapping network. GO terms are represented as nodes, and the strongest GO term pair-wise similarities are designated as edges in the graph. GO terms are grouped to illustrate the main biological processes characterizing the overlapping AD/T2DM networks. Complete lists of GO terms can be found in Supplementary Table 3.

**Figure 3:**
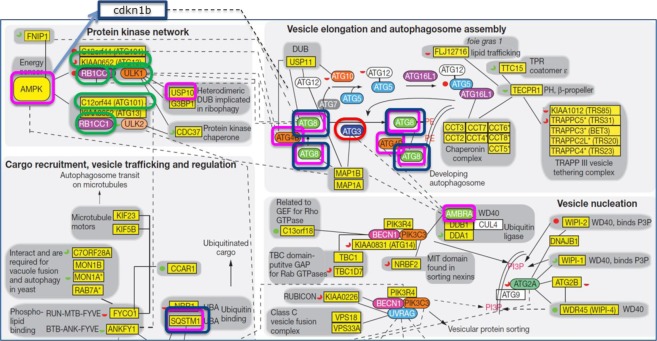
Integration of AD/T2DM overlapping network with human autophagy network (modified from Behrends, C., Sowa, M. E., Gygi, S. P. & Harper, J. W. Network organization of the human autophagy system. *Nature 466, 68–76 (2010))*. AD, T2DM and AD/T2DM genes are charted in the functional integration of the autophagy interaction network. In blue are shown the genes validated in the two animal models of AD, in red the genes found in the AD network, in green the genes located in the T2Dm network and in pink the AD/T2DM genes.

In the overlapping AD/T2DM network, a strong involvement of autophagy was further confirmed (Figs 2 and 3). In addition, apoptosis and Notch signalling were strongly involved together with oxidative stress and inflammation functions (Fig. 2, Supplementary Table S1). Functional Pathway enrichment analysis confirmed the GO terms findings and indicated a role also for FoxO, Wnt, estrogen, and leptin signalling pathways (Table 3).

**Table 3:** Results of the pathway analysis of the AD/T2DM overlapping network.

2.93E-09	5.29E-08	Signaling by NOTCH	Reactome
3.90E-09	6.69E-08	FoxO signaling pathway - Homo sapiens (human)	KEGG
7.18E-09	1.17E-07	Thyroid hormone signaling pathway - Homo sapiens (human)	KEGG
1.14E-07	1.53E-06	Macroautophagy	Reactome
1.59E-07	2.05E-06	Cellular Senescence	Reactome
1.15E-06	1.27E-05	Signaling by Wnt	Reactome
7.82E-06	6.79E-05	Regulation of autophagy - Homo sapiens (human)	KEGG
4.92E-05	0.000301243	Wnt signaling pathway - Homo sapiens (human)	KEGG
6.25E-05	0.000360329	Notch signaling pathway - Homo sapiens (human)	KEGG
0.000211397	0.0010285	Estrogen signaling pathway - Homo sapiens (human)	KEGG
0.000262185	0.001207105	Regulation of lipid metabolism by Peroxisome proliferator-activated receptor alpha (PPARalpha)	Reactome
0.000293703	0.001343203	Signaling by Interleukins	Reactome
0.000610253	0.002568306	Oxidative Stress Induced Senescence	Reactome
0.000693496	0.002848733	Central carbon metabolism in cancer - Homo sapiens (human)	KEGG
0.000864821	0.00342929	Neurotrophin signaling pathway - Homo sapiens (human)	KEGG
0.000990053	0.003773201	Signalling by NGF	Reactome
0.003406249	0.009736195	Innate Immune System	Reactome
0.009010442	0.018960624	Signaling by Leptin	Reactome

The main functional annotation analysis results of the two networks separately and of the overlapping AD/T2DM network are summarized in Figs 2 and 3, which highlights the central role discovered for the autophagic pathway.

### Validation in animal models of disease

**Figure 4:**
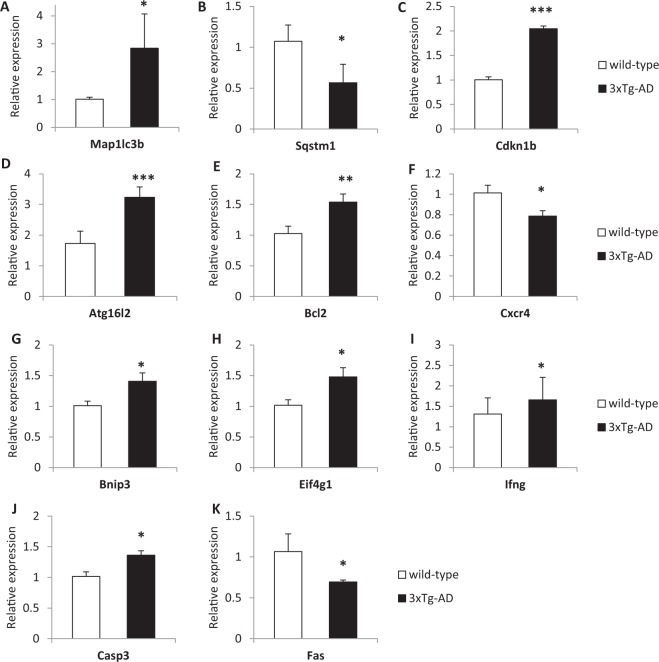
Altered mRNA expression levels autophagy-related genes in the pre-frontal cortex of 3xTg-AD (PS1tm1Mpm, human APPswe and TauP301L) homozygus mice with respect to wild-type mice. (**A**) Map1lc3b; (**B**) Sqstm1; (**C**) Cdkn1b; (**D**) Atg16l2; (**E**) Bcl2; (**F**) Cxcr4; (**G**) Bnip3; (**H**) Eif4g1; (**I**) Ifng; (**J**) Casp3; (**K**) Fas. ***p < 0.001; **p < 0.01; *p < 0.05.

Network analyses indicated that autophagy could be among the biological mechanisms underpinning both AD and T2DM, thus we performed a focused gene expression investigation in two animal models of Alzheimer’s disease with the aim of expanding the knowledge of the roles played by specific genes belonging to this pathway in the pathophysiology of this neurodegenerative disease. In the pre-frontal cortex of 3xTg-AD mice, a differential expression was detected for 11 genes involved in the autophagic pathway (Fig. 4, Table 4). The evidence of dysregulation was particularly strong for Cyclin-Dependent Kinase Inhibitor 1B (Cdkn1b) and for Autophagy Related 16-Like 2 (Atg16l2), which both showed much higher expression levels in 3xTg-AD mice with respect to wild-type controls (Fig. 4).

**Table 4:** Summary table of the gene expression results in the prefrontal cortex of two AD animal models in comparison with AD and T2DM networks. +  = p-value ≤ 0.05; +  + corrected p-value ≤ 0.05.

Gene symbol	In AD or T2DM network	3xTG vs. wild type	ApoE3 vs. ApoE4
ATG3	AD		+
GABARAPL2	AD/T2DM		+
MAP1LC3B	AD/T2DM	+	+
SQSTM1	AD/T2DM	+	
Cdkn1b		++	
Atg16l2		++	
Bcl2		+	
Cxcr4		+	
Bnip3		+	
Eif4g1		+	
Ifng		+	+
Casp3		+	
Fas		+	
Tnf			+
Ins2			++
Gaa			+
Ctss			+
Dram1			+
Atg7			+
B2m			+
Rps6kb1			+
Ctsb			+
Ctsd			+

**Figure 5:**
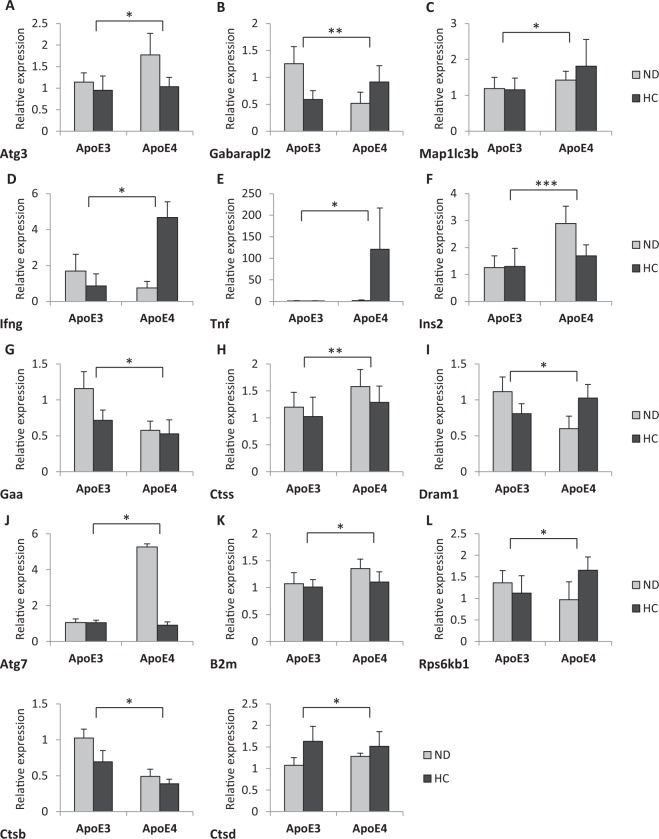
Altered mRNA expression levels autophagy-related genes in the pre-frontal cortex of hApoE TR mice expressing human ApoE3 or ApoE4 fed with either normal diet (ND) or high carbohydrate diet (HC). (**A**) Atg3; (**B**) Gabarapl2; (**C**) Map1lc3b (**D**) Ifng; (**E**) Tnf; (**F**) Ins2; (**G**) Gaa; (**H**) Ctss; (**I**) Dram1; (**J**) Atg7; (**K**) B2m; (**L**) Rps6kb1; (**M**) Ctsb; (**N**) Ctsd. ***p < 0.001;**p < 0.05; *p < 0.05.

In the ApoE4 targeted replacement (TR) mice, 14 genes involved in the autophagic process showed altered expression as compared to the ApoE3 mice (Fig. 5, Table 4). Among those, higher insulin gene expression in the ApoE4 genotype is highlighted (Fig. 5). In addition, mice belonging to each genotype were fed for six months with a high carbohydrate diet to stress the metabolism or with a normal diet and the alteration in gene expression was assessed. The results showed an overall modest impact of the high carbohydrate diet. In particular, Fas gene expression was up-regulated in the high carbohydrate diet vs. normal diet in the ApoE3 genotype, whereas Dram1 was strongly expressed in the high carbohydrate diet vs. normal diet in the ApoE4 genotype.

## Discussion

There is an ongoing debate about the role of T2DM in contributing to AD pathogenesis. Epidemiological and biological evidence strongly suggests the importance of T2DM components in AD pathology^4,6^, thus in the present study we investigated the molecular basis of this comorbidity applying a systems biology approach to transcriptomic data of human post mortem brains of AD or T2DM affected individuals.

AD and T2DM specific networks were built using a two-step workflow consisting in the identification of transcriptomic signatures followed by functional analysis based on network analysis. This approach allowed the identification of the molecular mechanisms underlying T2DM and AD and the commonalities and differences between the two.

AD network pathway enrichment confirmed previous findings supporting a role for inflammation in the pathophysiology of the disease. In the onset of inflammatory process the overexpression of specific interleukins such as (IL)-1 plays a relevant role producing many reactions in a loop that cause dysfunction and neuronal death^24^. In our AD network, but also in the T2DM network, we found the interleukin 7 receptor (IL7R). This interleukin receptor has been mostly known as a key regulator of T lymphocyte development and homeostasis, but recently it has been associated also to obesity and insulin resistance^25^, suggesting that this could possibly represent the molecular link between inflammatory pathways and metabolic alterations. Immune response and regulation of metabolism are highly integrated processes, dysfunction of which can lead to a cluster of chronic metabolic disorders including insulin resistance.

The analysis of the AD/T2DM overlapping network showed that the autophagic pathway is a crucial component in the pathophysiology of both diseases. Autophagy is a major catabolic pathway that allows recycling of cellular constituents into bioenergetic and biosynthetic materials for maintenance of homeostasis. In particular, neuronal autophagy is essential for synaptic plasticity, anti-inflammatory function in glial cells, oligodendrocyte development, and myelination process^26,27^. Indeed, a wealth of recent data indicated that autophagy plays a major role in the pathogenesis of AD^28,29^. These findings are particularly relevant since our open approach was not based on a pre-defined hypothesis. Dys-regulations in autophagy-related genes and proteins has been demonstrated in animal models of disease and in patients^30^; moreover, genetic studies reported associations between several autophagy genes and AD^31^. It has been proposed that the molecular mechanism underlying the association is based on the function of autophagy as a major pathway for the clearance and degradation of aggregated and toxic proteins^29,32^. Moreover, impaired mitophagy has also been associated to AD; mitophagy is the process by which cells detect and remove damaged mitochondria by an authophagic mechanism. Thus, impaired mitophagy leads to reduced cellular energy levels, increased reactive oxygen species, and impaired neuroplasticity^33,34^. It is likely that both autophagic removal of abnormal proteins and mitophagy-dependent elimination of damaged mitochondria are needed to prevent the development of AD. Consequently, although many components still need to be elucidated, the identification of the autophagic pathway as a fundamental component of AD aetiology is indicating a new direction for the discovery of potential targets for therapeutic intervention which could generate disease modifying medicines^32,35,36^, also addressing the transcriptional control of the process^32,37^. A number of links also exist between T2DM and autophagy, since the latter is the main physiological mechanism activated by energy restriction and it could have relevance in pancreatic β-cell homeostasis. Available evidence suggests that autophagy deficiency in pancreatic β cells could influence the progression from obesity to T2DM and it could contribute to insulin resistance in target organs^38–40^. The principal players include the central function exerted by AMPK, which is both a main regulator of energy metabolism and autophagy and a target of antidiabetic agents^41^; the autophagy-regulator mTOR pathway, which is involved in both glucose homeostasis and insulin resistance^42^; mitochondria dysfunction^43,44^; and the modulation of inflammation and immune cell function^45^. Among the genes highlighted in the network analysis, four were also confirmed in the Alzheimer’s disease animal models: ATG3, GABARAPL2 (ATG8), MAP1LC3B, and SQSTM1. As shown in Fig. 5, two of them GABARPL2 (ATG8) and MAP1LC3 are strongly involved in vesicle elongation and autophagosome assembly. ATG8 participates in cargo recruitment to the autophagosome^46^. Also SQSTM1 (p62) a multifunctional scaffolding protein commonly found in ubiquitinated inclusion bodies^47^ is a crucial protein in vesicle trafficking and regulation. The scaffolding protein p62 recruits autophagic protein substrates to LC3-bound autophagosomal membranes and it is associated with neuropathological inclusions in several neurodegenerative disorders, including AD.

SQSTM1 (p62) immunoreactivity is associated with neurofibrillary tangles and is involved in tau degradation and it has been shown that it regulates Aβ turnover. In addition SQSTM1 (p62) overexpression decreases Aβ by an autophagy-mediated mechanism and it rescues cognitive function and reduces Aβ pathology in APP/PS1 mice, a widely used animal model of AD^48^. Thus, it may play an essential role in eliminating senescent or damaged proteins and organelles in neurons and in β-cells, thereby protecting these cells from death. Alteration at this stage of sequestration of autophagic cargo, which has been shown to be selective^49^, could predispose the onset of both diseases. In support of this hypothesis SQSTM1 (p62) and MAP1LC3B are lower in Alzheimer patients carrying the ɛ4/ɛ4 genotype, which is one of the strongest predisposing factors in AD^50^.

The validation of the role of autophagy-related genes in animal AD models confirmed the importance of this pathway and suggested that specific genes are especially dysregulated in the disease model groups. Among them, Cyclin-Dependent Kinase Inhibitor 1B (Cdkn1b) and Autophagy Related 16-Like 2 (Atg16l2) showed higher expression levels in 3xTg-AD mice.

Cdkn1b is a cyclin-dependent kinase inhibitor able to block cell cycle progression into the proliferative stage. In addition to this first-discovered function, subsequent data showed that Cdkn1b is involved in other cellular processes, including autophagy. In particular, Cdkn1b is required to induce autophagy in nutrient-depleted cells through its stabilization mediated by AMPK-mediated phosphorylation^51^. Cdkn1b ability to promote autophagy by directly increasing autophagy flux with or without nutrient withdrawal was demonstrated by Sun *et al*.^52^ in differentiated cardiomyocytes, suggesting that the same could happen in other cell types lacking proliferative capacity such as neurons. However, previous studies have reported increased expression of cell cycle proteins in neurons of AD patients and it has been put forward that neuronal cell cycle re-entry may be involved in AD pathology^53^. The significance of this process in AD pathology is not clear; it has been proposed that cell cycle proteins in post-mitotic neurons may contribute to DNA repair and neuroplasticity^53^. Cdkn1b involvement in AD pathology is supported by data showing altered levels in patient-derived lymphoblasts^54^ and in post-mortem studies^55^, although in opposite directions. Therefore, our results provide further support to the significance of Cdkn1b, although our data do not allow drawing definite conclusions about the mechanism of its action.

Atg16l2 is a poorly characterised gene and its function in the autophagic pathway is incompletely understood. Atg16-like proteins interact with Atg5 and Atg12 to form a complex, the E3 ligase, which produces LC3 II, a required step in the formation of the autophagosome. While it is established that this function can be accomplished by Atg16l1, its homologue Atg16l2 is reported to be able to form the complex, but it does not seem to participate in autophagosome formation, at least in starvation-induced autophagy^56^. On the other hand, ATG16L2 altered expression or genetic polymorphisms have been associated to multiple sclerosis^57,58^, Crohn disease^59,60^, and systemic lupus erythematosus^61^, suggesting that this isoform performs a distinctive, critical function in autophagy. Since Atg16l2 can form hetero-oligomers with Atg16l1, it is possible that the presence of Atg16l2 in the complex may modulate the efficiency of autophagy or that Atg16l2 may be involved in a specialised type of autophagy^58^. In agreement with this notion, Li *et al*.^62^ discovered that Atg16l2 interacts with IKKα in pancreatic cells, acting as an adaptor protein and exerting an essential role in autophagy in these cells. The reported associations with autoimmune diseases suggest the possibility that Atg16l2 may also perform a specific function in the regulation of immune responses. In agreement with our data showing increased levels in a model of a neurodegenerative disorder, Sittler *et al*. detected increased ATG16L2 immunoreactivity in brain regions of Machado‐Joseph disease patients^63^.

In conclusion, this study demonstrated that a systems biology approach can be a powerful tool to identify common pathophysiological mechanisms underpinning AD and T2DM. The analysis of human brain transcriptomic data by identification of transcriptional signatures followed by network expansion and functional annotation analysis revealed that a central role is played by autophagy. The relevance of the autophagic pathways was validated in diseases models, allowing the identification of specific genes bearing a significant contribution to the pathway dysregulation.

## Methods

### Experimental design

A schematic representation of the study workflow is shown in Fig. 1. A systems biology workflow was applied to the transcriptomic data of AD and T2DM affected subjects. Gene expression data were analysed with a recently developed algorithm for signature-based clustering of expression profiles to devise a transcriptional signature able to discriminate individuals belonging to the disease groups from controls. We identified a transcriptional signature for each of the two diseases, and performed network analysis in order to expand the list of signature genes with the help of protein-protein interactions from the literature. The resulting enlarged list was characterized through functional annotation in the form of gene ontologies and canonical pathways. The AD and T2DM networks and functional annotation results were then compared. The significant role for the autophagic pathway discovered by the system biology approach was then validated by transcriptional analysis of selected genes in two relevant animal models of AD coupled with a metabolic challenge.

### Transcriptomic data

The microarray data set was downloaded from the GEO (GSE36980). The original dataset contains gene expression profiles of grey matter from post-mortem brains of AD (n = 16) and non-AD controls (n = 23) donated for the Hisayama study^64^. For our study we used the profiles for which information on diabetes mellitus status could be derived from the Hokama *et al*. paper^64^. The assessment of AD pathology was conducted according to the Consortium to Establish a Registry for Alzheimer’s Disease (CERAD) guidelines^65^ and the Braak stage^66^. Clinical data related to T2DM or pre-diabetes were collected as described elsewhere^67^.

### Transcriptomic signature identification

The transcriptional signatures have been identified by means of a rank-based classification method previously implemented in the web-tool SCUDO^68,69^. Briefly, an average expression profile was computed and then each of the 57 profiles considered in this study was divided by it. A preliminary feature selection step was performed, consisting in a pairwise t test (threshold: p-value < 0.01, genes selected were all those appearing in at least one of the two lists resulting from the following two comparisons: i) T2DM vs controls subjects, and ii) AD vs controls subjects). Then our classification method was applied; the method ranks the filtered genes by expressions level separately for each sample, and then it produces a set of subject-specific signatures, where each signature is the list of the first n1 and the last n2 genes in the ranking (n1 and n2 have the same value for all subjects and are parameters of the method). An all-to-all signature comparison was then carried out using a distance metric based on an enrichment score, resulting in a distance matrix that systematically quantifies the degree of similarity between each pair of subjects. The k^th^ percentile of values from the distance matrix was then used to build a map of individuals. The map is in the form of a graph where the nodes correspond to signatures/subjects and the length of a connecting edge encodes the level of similarity between the connected nodes (short edge = high similarity; no edge = negligible similarity). Finally, a consensus gene signature was extracted for each group of subjects, which collected all the genes included in at least one subject-specific signature for that group. The SCUDO method was used to perform a 4-way classification (four groups: T2DM controls, non-T2DM controls, T2DM AD, and non-T2DM AD subjects) with the following parameter values: n1 = 100, n2 = 100, k = 10 i.e. bottom 10% of distances was used to generate the graph. Only two groups were further studied (T2DM controls and non-T2DM AD subjects), and their consensus signatures extracted for further analysis. The consensus signatures for the T2DM and the AD groups included 200 probe names each, which produced two lists of 126 and 108 genes (T2DM and AD, respectively) after the probe name to gene symbol translation. The translation was performed manually using the annotation table for the Affymetrix Human Gene 1.0 ST Array platform downloaded from GEO.

### Network enrichment

The networks of AD and T2DM were extracted from the Biogrid Database^70^ using the AD or T2DM disease signatures as seed genes and protein-protein interactions for Homo Sapiens. BioGRID is one of the most comprehensive protein interaction databases, containing more than 1,400,000 interactions curated from both high-throughput datasets and individual focused studies. From the raw data from BioGrid, we considered only non-redundant human interactions. Each unique combination of interactors, protein A and protein B, are counted as a single interaction, regardless of directionality, experimental system and publication. The genetic inferred interactions were filtered out to remain only physical interactions. We extracted direct 1-step interactions of the AD and T2DM genes to construct two corresponding networks, AD network and T2DM network. The network of AD consists of 620 genes and 889 interactions and the network of T2DM contains 599 genes and 869 interactions. Both of two networks are not so dense, but rather centralized with some big hub of proteins.

### Functional annotation analysis

The gene lists obtained from network enrichment analysis were used to extract the most representative GO biological process terms. For identifying and visualizing enriched GO terms, we used GOrilla and REVIGO tools^71,72^. Hypergeometric distribution was applied to test GO term enrichment, and an adjusted p-value (FDR) threshold of 0.05 was selected. Pathway analysis was performed using ConsensusPathDB^73^.

### Animals

3xTg-AD (PS1tm1Mpm, human APPswe and TauP301L) homozygous mice^74^ and the respective C57-derived wild type animals were kindly provided by prof A. Genazzani, University of Eastern Piedmont “Amedeo Avogadro”, Italy. Forty-eight weeks old mice of mixed gender were investigated (n = 6/group). The mice were housed in groups of 4–6 in ventilated cages (Tecniplast, Italia) with lights on from 7.00 am to 7.00 pm, 22 ± 2◦C temperature and 65% humidity with water and food ad libitum.

hApoE TR mice expressing human apoE3 and apoE4 under the control of the murine apoE regulatory sequences and on a C57BL/6 J background, were purchased from Taconic Farms (USA). Colonies of hApoE3 (B6.129P2-Apoetm2(APOE*3)Mae N8) and hApoE4 (B6.129P2-Apoetm3(APOE*4)Mae N8) TR mice were maintained by homozygous breeding^75^. The pups were weaned at 3 weeks of age and fed for 6 months with either normal diet (ND) (Mucedola s.r.l., Milano, Italy) or high carbohydrate diet (HCD) containing 70% carbohydrates (Mucedola s.r.l.). Adult male mice were selected for the study (n = 6/group).

All experiments were carried out in accordance with Directive 86/609/EEC and approved by the Animal Welfare Committee at the University of Bologna (PROT. n. 15-IX/9). Care was taken to minimize the number of experimental animals and to take measures to limit their suffering.

### RNA extraction and retrotranscription

Mice were sacrificed by cervical dislocation under gaseous anesthesia (5% isoflurane in 1 L/min oxygen/nitrous oxide), pre-frontal cortices were dissected and stored at −80 °C. Total RNA was extracted by using the Aurum Total RNA Fatty and Fibrous Tissue Kit (Bio-Rad, Hercules, USA) following manufacturer’s instructions. RNA was quantified by UV spectrophotometry in a NanoDrop 2000c UV-Vis Spectrophotometer (ThermoFisher Scientific); RNA purity was confirmed by a ratio value OD260/OD280 ≥ 2. RNA integrity was verified by 1% agarose electrophoresis. cDNA was synthesized by using the iScript Advanced cDNA synthesis Kit (Bio-Rad) following manufacturer’s instructions.

### Quantitative PCR

Quantitative real-time PCR reactions by Sybr Green technology were performed in a CFX Real-Time PCR System (Bio-Rad) with 50 ng cDNA and Sso Advanced Universal SYBR Green Supermix in Autophagy (SAB Target List) H96 plates (Bio-Rad, cat # 10034108) containing primers for the following genes: Actin, Atg3, Atg9b, Bid1, Ctsd, Esr1, Hdac1, Ins2, Nfkb1, Rb1, Tnf, GAPDH, Atg4a, B2m, Bnip3, Ctss, Fadd, Hdac6, Irgm1, Npc1, Rgs19, Tnfsf10, Hprt, Ambra1, Atg4b, Bad, casp3, Cxcr4, fas, Hgs, Lamp1, Pik3c3, Rps6kb1, Trp53, App, Atg4c, Bak1, Casp8, Dapk1, Gaa, Hsp90aa1, Map1lc3a, Pik3cg, Snca, Ulk1, Atg1, Atg4d, Bax, Cdkn1b, Dram1, Gabarap, Hsp90ab1, Map1lc3b, Pik3r4, Sqstm1, Ulk2, Atg12, Atg5, Bcl2, Cdkn2a, Dram2, Gabarapl1, Htt, Mapk14, Prkaa1, Tgfb1, Uvrag, Atg16l1, Atg7, Bcl2l1, Cln3, Eif2ak3, Gabarapl2, Ifng, Mapk8, Pten, Tgm2, Wipi1, Atg16l2, Atg9a, Becn1, Ctsb, Eif4g1, Gusb, Igf1, Mtor, Rab24, Tmem74, Tbp. To provide quantification, a threshold cycle (Ct) number was defined in the early logarithmic phase of the amplification plot and the relative expression of gene transcripts was calculated by the Delta-Delta Ct (DDCt) method and converted to relative expression ratio (2-^DDCt^) for statistical analysis^76^. All data were normalized to the geometric average of the endogenous reference genes glyceraldehyde-3-phosphate dehydrogenase (GAPDH), Actin (Act), and TATA box binding protein (Tbp) expression^77^. A dissociation curve was built in the 60–95 °C range to evaluate amplification product specificity.

### Statistical analysis of real-time PCR data

The quantitative real-time PCR data was processed in R using the RankProd package^78^. The RP function from the package was used with its default values with n = 10000 permutations for computing the empirical distributions. RankProduct is a rank-based method capable of identifying differentially expressed genes with high sensitivity even in the case of a modest number of profiles. Moreover, being based on ranks the method makes no assumptions on the distribution of expression values, and it is robust to real-time PCR normalization errors.

## Supplementary information

Supplementary Figure 1

Supplementary Table 1

## Data Availability

The datasets generated and analysed during the current study are available from the corresponding author on reasonable request.
