# Supplementary material for: Cross-disease analysis of Alzheimer’s disease and type-2 Diabetes highlights the role of autophagy in the pathophysiology of two highly comorbid diseases
Source: Sci Rep. 2019 Mar 8;9:3965. doi: 10.1038/s41598-019-39828-5 (PMC6408545; doi:10.1038/s41598-019-39828-5)
Supplement: Supplementary file 1 — Supplementary Figure 1 [file 41598_2019_39828_MOESM1_ESM.pdf]

Laura Caberlotto, T.-Phuong Nguyen, Mario Lauria, Corrado Priami, Roberto Rimondini, Silvia Maioli, Angel Cedazo-Minguez, Giulia Sita, Fabiana Morroni, Mauro Corsi, Lucia Carboni.

Cross-disease analysis of Alzheimer's disease and type-2 diabetes highlights the role of autophagy in the pathophysiology of two highly comorbid diseases

Supplementary Figure 1.

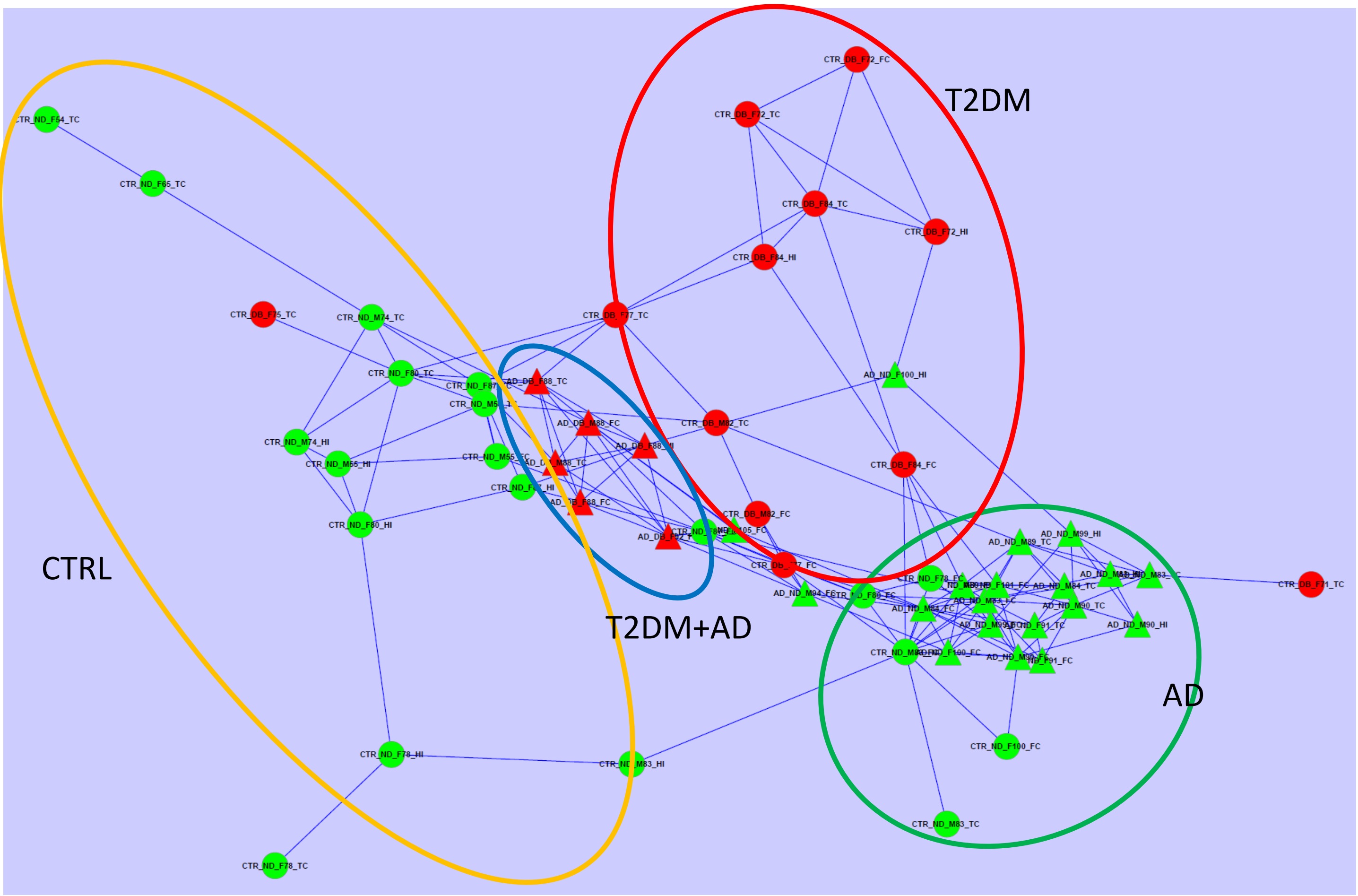

Supplementary Figure 1. Map of profiles according to their transcriptional signature similarity. The map is in the form of a graph where the nodes correspond to profiles and the length of a connecting edge encodes the level of similarity between the connected nodes (short edge = high similarity; no edge = negligible similarity). The SCUDO method was used to perform a 4-way classification based on the following four groups: T2DM controls, non-T2DM controls, T2DM AD, and non-T2DM AD subjects. Legend: *red:* T2DM, *green:* non-T2DM; *triangles:* AD subjects; *circles: non-AD subjects*.
